# PARP inhibitor-based first-line maintenance therapy for newly diagnosed advanced ovarian cancer across molecular subgroups: an updated systematic review and network meta-analysis

**DOI:** 10.3389/fonc.2026.1884407

**Published:** 2026-09-18

**Authors:** Juntao Feng, Xiaomei Qin, Xiaoliang Mo

**Affiliations:** Department of Gynecology and Obstetrics, The First Affiliated Hospital of Guangxi Medical University, Nanning, China

**Keywords:** bevacizumab, BRCA mutation, first-line maintenance therapy, homologous recombination deficiency, molecular subgroup, network meta-analysis, ovarian cancer, PARP inhibitor

## Abstract

**Background:**

PARP inhibitor-based maintenance has become central to first-line treatment of newly diagnosed advanced ovarian cancer. With expanding therapeutic options, treatment selection increasingly relies on BRCA mutation and homologous recombination deficiency (HRD) status. The 2026 NCCN Guidelines emphasize biomarker-directed maintenance; however, comparative evidence across molecular subgroups remains limited. We conducted an updated network meta-analysis to compare efficacy and safety of PARP inhibitor-based first-line maintenance strategies.

**Methods:**

We searched major databases and conference proceedings to 1 April 2026 for randomized controlled trials evaluating first-line maintenance in newly diagnosed advanced epithelial ovarian, fallopian tube, or primary peritoneal cancer. Eligible interventions included PARP inhibitor monotherapy, PARP inhibitor plus antiangiogenic therapy, antiangiogenic monotherapy, and placebo. Primary analyses focused on progression-free survival (PFS) in BRCA-mutated and HRD-positive populations; secondary and exploratory analyses covered intention-to-treat, HRD-positive non-BRCA-mutated, and homologous recombination proficient populations. Safety outcomes included grade ≥3 adverse events and treatment discontinuation. A frequentist random-effects model was applied.

**Results:**

Seven trials involving 3,930 patients were included. PARP inhibitor-based maintenance significantly improved PFS versus placebo, with the strongest benefit in BRCA-mutated and HRD-positive populations. PARP inhibitor monotherapy also significantly improved PFS in the HRD-positive non-BRCA-mutated population, supporting clinical relevance of HRD status beyond BRCA mutation alone. Benefit in the intention-to-treat population was smaller than in biomarker-selected populations. Evidence of benefit in the homologous recombination proficient population was inconsistent. Indirect comparisons showed no statistically significant superiority of one active regimen over another. Within the connected network, no statistically significant incremental PFS benefit was established for adding antiangiogenic therapy to PARP inhibition; this comparison was limited by the restricted evidence base for combination therapy and the inability to incorporate PAOLA-1 into the primary network because of network disconnection. No statistically significant evidence indicated that frontline bevacizumab exposure modified the relative PFS benefit. Sensitivity analyses yielded results consistent with the primary analysis. PARP inhibitor monotherapy increased grade ≥3 adverse events versus placebo; comparative safety of combinations remained limited.

**Conclusions:**

This network meta-analysis supports biomarker-directed PARP inhibitor-based first-line maintenance in advanced ovarian cancer, with the greatest PFS benefit in BRCA-mutated and HRD-positive populations. No active regimen demonstrated statistically significant superiority within the connected network. The incremental benefit of adding antiangiogenic therapy to PARP inhibition remains uncertain because connected-network evidence for combination strategies was limited and PAOLA-1 could not be incorporated into the primary network. These findings should not be interpreted as evidence against the clinical value of olaparib plus bevacizumab or combination maintenance strategies in general. Treatment selection should integrate molecular status, prior bevacizumab exposure, safety, treatment availability, and patient preference.

**Systematic review registration:**

https://www.crd.york.ac.uk/PROSPERO/, identifier CRD420261382266.

## 1 Introduction

Ovarian cancer remains one of the most lethal gynecologic malignancies worldwide, with most patients presenting with advanced-stage disease at diagnosis (1, 2). Although primary cytoreductive surgery followed by platinum-based chemotherapy remains the standard initial treatment for newly diagnosed advanced ovarian cancer, most patients ultimately experience disease recurrence despite achieving an initial complete or partial response. Therefore, optimizing first-line maintenance therapy after response to platinum-based chemotherapy has become a central strategy for prolonging disease control in this setting (3, 4).

Poly(ADP-ribose) polymerase (PARP) inhibitors have substantially reshaped the frontline maintenance landscape. By exploiting synthetic lethality, PARP inhibition is particularly effective in tumors with impaired homologous recombination repair, especially those harboring BRCA1/2 mutations or broader homologous recombination deficiency (HRD) (5–7). Several pivotal randomized trials have shown that PARP inhibitor maintenance significantly prolongs progression-free survival (PFS) in the first-line setting, with the greatest benefit generally observed in BRCA-mutated (BRCAm) and HRD-positive disease (8–10). In contrast, benefit in homologous recombination proficient (HRP) tumors has been less consistent and remains clinically controversial.

Antiangiogenic therapy has also been incorporated into first-line treatment and maintenance strategies for selected patients (11). Combining PARP inhibition with antiangiogenic therapy has a biologic rationale, as vascular endothelial growth factor pathway inhibition may induce tumor hypoxia and suppress homologous recombination repair, thereby increasing sensitivity to PARP inhibition (12, 13). Clinical evidence for PARP inhibitor plus antiangiogenic maintenance has expanded available treatment options. However, whether combination therapy provides incremental benefit over PARP inhibitor monotherapy across molecularly defined subgroups remains uncertain, particularly given its potential to increase treatment burden and toxicity.

As the number of available maintenance strategies has expanded, treatment selection has become increasingly complex. The 2026 National Comprehensive Cancer Network (NCCN) Guidelines for ovarian, fallopian tube, and primary peritoneal cancers further emphasize a biomarker-directed approach to first-line maintenance therapy (14), in which BRCA mutation status, HRD status, and prior or concurrent bevacizumab exposure are key determinants of treatment choice. However, direct comparative evidence among active maintenance regimens remains limited. This gap is especially relevant when selecting between PARP inhibitor monotherapy and PARP inhibitor–antiangiogenic combinations, and when applying maintenance therapy to clinically important subgroups such as HRD-positive non-BRCAm and HRP disease.

Previous meta-analyses have confirmed the overall benefit of PARP inhibitor maintenance in newly diagnosed advanced ovarian cancer (15), More recently, Zhang et al. conducted a network meta-analysis evaluating multiple PARP inhibitors in this setting, providing valuable comparative estimates across molecularly defined subgroups (16). However, several important gaps remain unaddressed. First, their literature search was limited to January 2025, predating the reporting of FZOCUS-1, a phase III randomized controlled trial evaluating fuzuloparib—a PARP inhibitor not previously incorporated into any network meta-analysis. Second, the clinically distinct subpopulation of HRD-positive non-BRCA-mutated patients, who represent a group with considerable therapeutic uncertainty, was not separately analyzed. Third, the potential effect-modifying role of frontline bevacizumab background therapy on the comparative efficacy of maintenance strategies has not been systematically explored. Fourth, the evolving biomarker-directed framework reflected in the 2026 NCCN Guidelines has not been incorporated as an analytical and interpretive context. Network meta-analysis enables simultaneous integration of direct and indirect evidence across multiple competing strategies within a unified statistical framework, and an updated analysis incorporating these advances is therefore warranted.

Accordingly, to address these evidence gaps, we conducted an updated systematic review and network meta-analysis of randomized controlled trials comparing first-line maintenance strategies for newly diagnosed advanced ovarian cancer, with a literature search extended to April 2026. Our analysis incorporated the most recently available trial data, including FZOCUS-1, and was conducted within the biomarker-directed framework of the 2026 NCCN Guidelines. We compared PARP inhibitor monotherapy, PARP inhibitor plus antiangiogenic therapy, and control across five molecularly defined populations: intention-to-treat, BRCAm, HRD-positive, HRD-positive non-BRCAm, and HRP. We additionally performed prespecified subgroup analyses stratified by frontline bevacizumab background to explore its potential role as an effect modifier of maintenance treatment efficacy.

## 2 Methods

### 2.1 Protocol and reporting

This systematic review and network meta-analysis was conducted according to the PRISMA-NMA statement (17). The protocol was registered in PROSPERO (CRD420261382266).

### 2.2 Eligibility criteria

Eligible studies were phase III randomized controlled trials, enrolling adults with newly diagnosed advanced epithelial ovarian, fallopian tube, or primary peritoneal cancer who had achieved complete or partial response after first-line platinum-based chemotherapy. Trials were included if they evaluated first-line maintenance therapy with a PARP inhibitor, either as monotherapy or in combination with anti-angiogenic therapy, and reported progression-free survival (PFS) hazard ratios (HRs) with 95% confidence intervals (CIs), or sufficient data for estimation, in the intention-to-treat (ITT) population and/or molecularly defined subgroups.

Eligible interventions included PARP inhibitor monotherapy and PARP inhibitor plus antiangiogenic therapy. Comparators included placebo, observation, antiangiogenic maintenance, or protocol-defined maintenance controls, including bevacizumab-containing controls where applicable. Trials of recurrent disease, platinum-sensitive or platinum-resistant relapse, non-epithelial ovarian cancer, exclusively early-stage disease, PARP inhibitors initiated concurrently with chemotherapy rather than as maintenance, or PARP inhibitor combinations in which the independent contribution of PARP inhibition could not be isolated were excluded. Non-randomized studies, single-arm studies, phase I studies, reviews, editorials, case reports, and duplicate reports without additional relevant data were also excluded.

When multiple reports of the same trial were available, the most complete and most recent dataset was used, with supplementary reports used for additional subgroup, follow-up, or safety information.

### 2.3 Search strategy and study selection

PubMed/MEDLINE, Embase, Web of Science Core Collection, and ClinicalTrials.gov were searched from database inception to 1 April 2026. Major oncology conference proceedings, including ASCO, ESMO, SGO, and IGCS, were also screened. Search strategies combined terms for epithelial ovarian, fallopian tube, and primary peritoneal cancer; PARP inhibitors; first-line maintenance therapy; newly diagnosed disease; platinum-based chemotherapy; randomized phase III trials; placebo-controlled or double-blind designs; and relevant trial names. Supplementary trial-name searches were performed to identify primary reports, updated analyses, subgroup analyses, and conference abstracts. Full database-specific search strategies are provided in **Supplementary Table S1**.

After duplicate removal, two reviewers (Juntao Feng and Xiaomei Qin) independently screened titles, abstracts, and full texts. Disagreements were resolved by discussion or consultation with a third reviewer (Xiaoliang Mo).

### 2.4 Data extraction and risk of bias

Two reviewers (Juntao Feng and Xiaomei Qin) independently extracted study characteristics, patient and disease features, biomarker definitions, HRD assay methods, frontline bevacizumab exposure, intervention and comparator regimens, follow-up duration, and efficacy and safety outcomes. PFS HRs and 95% CIs were extracted for the ITT, BRCA-mutated (BRCAm), HRD-positive, HRD-positive non-BRCAm, and homologous recombination proficient (HRP) populations when available. If several data cutoffs were reported, the most mature estimate for the same population and assessment method was used. PFS estimates based on blinded independent central review or investigator assessment were extracted according to the primary reporting method of each trial.

Safety data were extracted from the safety population defined in each trial and included grade ≥3 adverse events, adverse events leading to treatment discontinuation, and selected grade ≥3 hematologic toxicities. Risk of bias was assessed using the Cochrane Risk of Bias 2 tool (18).

### 2.5 Outcomes

The primary efficacy outcomes were PFS in the HRD-positive and BRCAm populations. Secondary efficacy outcomes were PFS in the HRD-positive non-BRCAm, intention-to-treat and HRP populations. The primary safety outcome was grade ≥3 adverse events; additional safety outcomes included treatment discontinuation due to adverse events and selected grade ≥3 hematologic toxicities.

PFS was analyzed using HRs, and safety outcomes using risk ratios (RRs). For PFS, HRs less than 1 favored the experimental regimen. For safety outcomes, RRs greater than 1 indicated higher toxicity risk.

### 2.6 Network geometry

Separate networks were constructed for each efficacy population and safety outcome. Nodes were defined at the regimen level rather than by drug class. In the primary connected efficacy networks, placebo served as the common comparator. The ITT, HRD-positive, and HRP networks included placebo, niraparib, rucaparib, senaparib, fuzuloparib, and fuzuloparib plus apatinib; the BRCAm network additionally included olaparib; and the HRD-positive non-BRCAm network included placebo, niraparib, rucaparib, and senaparib.

Direct active–active evidence was limited to the fuzuloparib plus apatinib versus fuzuloparib comparison in FZOCUS-1 (19). Other active-regimen comparisons were informed indirectly through placebo. PAOLA-1 (9) met the eligibility criteria for the systematic review. However, because both treatment arms received bevacizumab as mandatory background therapy and the control arm was bevacizumab-containing rather than placebo alone, PAOLA-1 was eligible for the systematic review but was not incorporated into the primary placebo-controlled connected network meta-analyses for efficacy because its treatment comparison formed a disconnected network component. In PAOLA-1, both treatment groups received bevacizumab as background therapy, and the comparison of olaparib plus bevacizumab versus bevacizumab plus placebo could not be linked to the other treatment nodes in the primary network. It was summarized narratively and considered only in descriptive contextual analyses where appropriate. PAOLA-1 was also excluded from the main safety network meta-analysis to maintain consistency with the placebo-controlled network structure.

### 2.7 Statistical analysis

Network meta-analyses were performed within connected networks using a frequentist framework. Log HRs and corresponding standard errors were used for PFS, and log RRs were used for dichotomous safety outcomes. Random-effects models were used given expected clinical and methodological heterogeneity across trials. Relative effects were reported with 95% CIs, and treatment rankings were summarized using P-scores as supportive measures.

Transitivity was considered by comparing key trial characteristics across comparisons, including biomarker-defined populations, HRD testing methods, bevacizumab exposure, comparator regimens, and PFS assessment methods. Because networks were largely placebo-centered and active–active evidence was sparse, indirect comparisons among active regimens were interpreted cautiously. Heterogeneity was assessed using τ² and, where applicable, I². Formal inconsistency testing was limited to networks with suitable closed loops; otherwise, consistency was judged through network structure and clinical comparability. Multi-arm trials were analyzed with methods accounting for correlation between comparisons from the same trial.

Analyses were conducted in R version 4.5.2 using the netmeta package version. Continuity corrections were applied for sparse dichotomous data or zero-event cells when required.

### 2.8 Exploratory and sensitivity analyses

An exploratory study-level effect-modification analysis assessed whether frontline bevacizumab background modified the PFS benefit of PARP inhibitor-based maintenance. Trials were categorized as requiring, allowing, or not allowing frontline bevacizumab. Pooled HRs were estimated within these categories, and differences were explored using meta-regression models with bevacizumab background as categorical and continuous variables. These analyses were considered hypothesis-generating because of limited trial numbers, ecological study-level covariates, and heterogeneity within categories.

Sensitivity analyses compared results with and without FZOCUS-1, which introduced fuzuloparib-based regimens and the only direct active-treatment comparison. Additional regimen-specific analyses evaluated individual PARP inhibitor monotherapies across the ITT, HRD-positive, BRCAm, HRD-positive non-BRCAm, and HRP populations where data were available.

### 2.9 Safety analysis

Safety network meta-analyses were performed for connected regimens with sufficient data, using placebo as the common comparator where possible. Grade ≥3 adverse events were defined according to the Common Terminology Criteria for Adverse Events (CTCAE) grading system, using the CTCAE version reported in each original trial. Because safety data for combination regimens were sparse and not consistently connected, comparative safety between PARP inhibitor monotherapy and PARP inhibitor–anti-angiogenic combinations was considered uncertain. Safety findings from disconnected bevacizumab-background comparisons, including PAOLA-1, were summarized descriptively rather than included in the primary safety network meta-analysis. Because safety outcomes were analyzed in the overall safety populations rather than molecular subgroups, safety network structures differed from efficacy subgroup networks.

## 3 Results

### 3.1 Study selection and characteristics of included trials

Seven phase III randomized, double-blind trials, involving 3930 patients, were included in the systematic review; six contributed to the primary placebo-controlled connected efficacy networks, whereas PAOLA-1 was excluded from the primary network for the reasons detailed in the Methods section.

Four trials were international multicenter studies [SOLO-1 (8), PAOLA-1 (9), PRIMA (10), and ATHENA-MONO (20)], and three were conducted in China [PRIME (21), FLAMES (22), and FZOCUS-1 (19)]. Interventions included PARP inhibitor monotherapy and PARP inhibitor plus anti-angiogenic therapy. Background frontline bevacizumab exposure varied across trials, with PAOLA-1 requiring bevacizumab, ATHENA-MONO including some prior bevacizumab exposure, and SOLO-1, PRIME, PRIMA, and FLAMES excluding frontline bevacizumab per protocol. In FZOCUS-1, 6.7% of patients had received bevacizumab during frontline chemotherapy. PFS was assessed by blinded independent central review in FZOCUS-1, PRIMA, PRIME, and FLAMES, and by investigator review in SOLO-1, PAOLA-1, and ATHENA-MONO. Study characteristics are presented in **Table 1**, and the study selection process is shown in **Figure 1**.

**Table 1:** Baseline characteristics of the included phase III randomized trials.

Trial	Year	Region	Arms	Total N	Intervention/comparator	Randomization	Bevacizumab background	PFS assessment	Molecular subgroups available
SOLO-1	2018	International	2	391	Olaparib vs placebo	2:1	No frontline bevacizumab	Investigator	BRCAm
PAOLA-1	2019	International	2	806	Olaparib + bevacizumab vs placebo + bevacizumab	2:1	Required bevacizumab	Investigator	ITT, HRD+, BRCAm, HRD+/non-BRCAm, HRP
PRIMA	2019	International	2	733	Niraparib vs placebo	2:1	No frontline bevacizumab	BICR	ITT, HRD+, BRCAm, HRD+/non-BRCAm, HRP
ATHENA-MONO	2022	International	2	538	Rucaparib vs placebo	4:1	Mixed/some prior bevacizumab	Investigator	ITT, HRD+, BRCAm, HRD+/non-BRCAm, HRP
PRIME	2023	China	2	384	Niraparib vs placebo	2:1	No frontline bevacizumab	BICR	ITT, HRD+, BRCAm, HRP
FLAMES	2024	China	2	404	Senaparib vs placebo	2:1	No frontline bevacizumab	BICR	ITT, HRD+, BRCAm, HRD+/non-BRCAm
FZOCUS-1	2026	China	3	674	Fuzuloparib + apatinib vs fuzuloparib vs placebo	2:2:1	6.7% received frontline bevacizumab	BICR	ITT, HRD+, BRCAm, HRP

BICR, blinded independent central review; ITT, intention-to-treat; HRD, homologous recombination deficiency; HRP, homologous recombination proficient; BRCAm, BRCA-mutated; PARPi, poly(ADP-ribose) polymerase inhibitor; PFS, progression-free survival.

N refers to the total randomized sample size of each trial.

**Figure 1:**
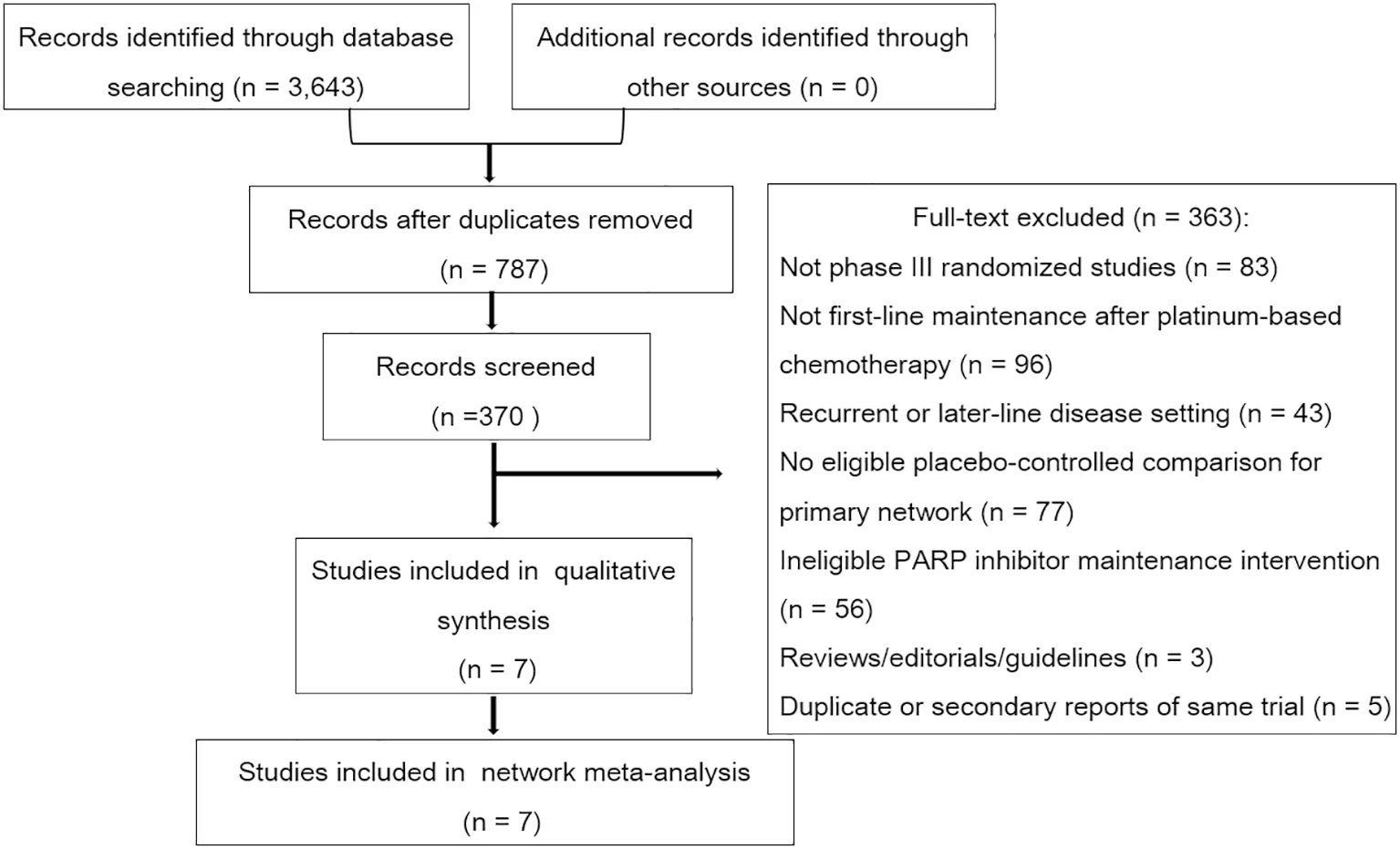
PRISMA flow diagram for study selection.

### 3.2 Risk of bias assessment

Risk of bias was evaluated using the Cochrane Risk of Bias 2 tool. All included trials were classified as low risk of bias overall, with no domain showing substantial concerns. Specifically, the randomization process, deviations from intended interventions, missing outcome data, outcome measurement, and selection of the reported result were all judged to be at low risk. The full domain-level assessment is shown in **Supplementary Figure S1**.

### 3.3 Network geometry

Separate PFS networks were generated for the ITT, HRD-positive, BRCAm, HRD-positive non-BRCAm, and HRP populations. Placebo was the common comparator across all connected networks.

The ITT, HRD-positive, and HRP networks included placebo, niraparib, rucaparib, senaparib, fuzuloparib, and fuzuloparib plus apatinib, whereas the BRCAm network additionally included olaparib. The HRD-positive non-BRCAm network was more limited, including only placebo, niraparib, rucaparib, and senaparib. No fuzuloparib or fuzuloparib plus apatinib data were available for this subgroup.

Direct active–active evidence was sparse. The only direct head-to-head comparison between active regimens was fuzuloparib plus apatinib versus fuzuloparib from FZOCUS-1; all other active treatment comparisons relied on indirect evidence through placebo. PAOLA-1 was excluded from these placebo-controlled networks as prespecified (see Methods for rationale). The network geometries are presented in **Figure 2**.

**Figure 2:**
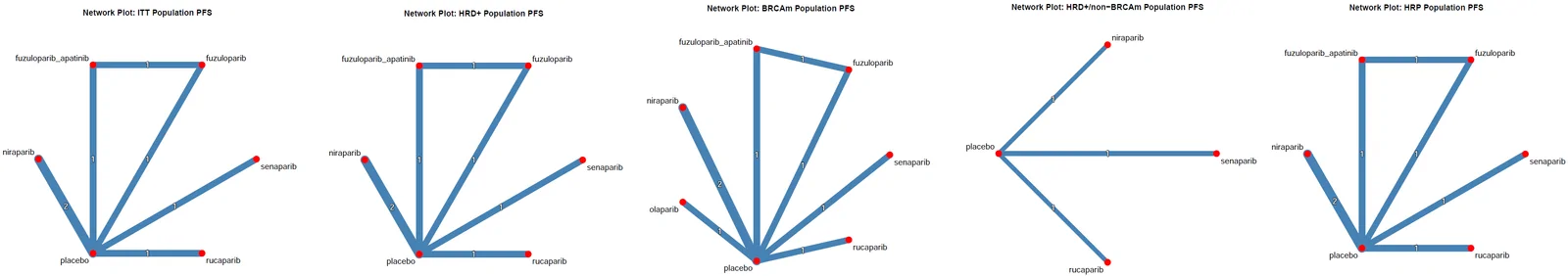
Network geometry for progression-free survival analyses. Network plots are shown for the ITT, HRD-positive, BRCAm, HRD-positive non-BRCAm, and HRP populations, corresponding to the titles displayed above each plot from left to right. Nodes indicate treatment regimens, and edges indicate available direct comparisons. Numbers on the edges represent the number of trials informing each direct comparison. Placebo was the common comparator across the connected networks.

### 3.4 Primary efficacy outcomes

#### 3.4.1 HRD-positive population

In the HRD-positive connected placebo-controlled network (**Figure 3A**), all evaluated PARP inhibitor-based maintenance regimens significantly improved PFS compared with placebo. The estimated HRs versus placebo were 0.67 for fuzuloparib plus apatinib (95% CI, 0.48–0.94), 0.62 for fuzuloparib (95% CI, 0.44–0.86), 0.47 for rucaparib (95% CI, 0.31–0.72), 0.45 for niraparib (95% CI, 0.36–0.57), and 0.36 for senaparib (95% CI, 0.22–0.60). Based on P-scores, senaparib ranked highest, followed by niraparib, rucaparib, fuzuloparib, fuzuloparib plus apatinib, and placebo.

**Figure 3:**
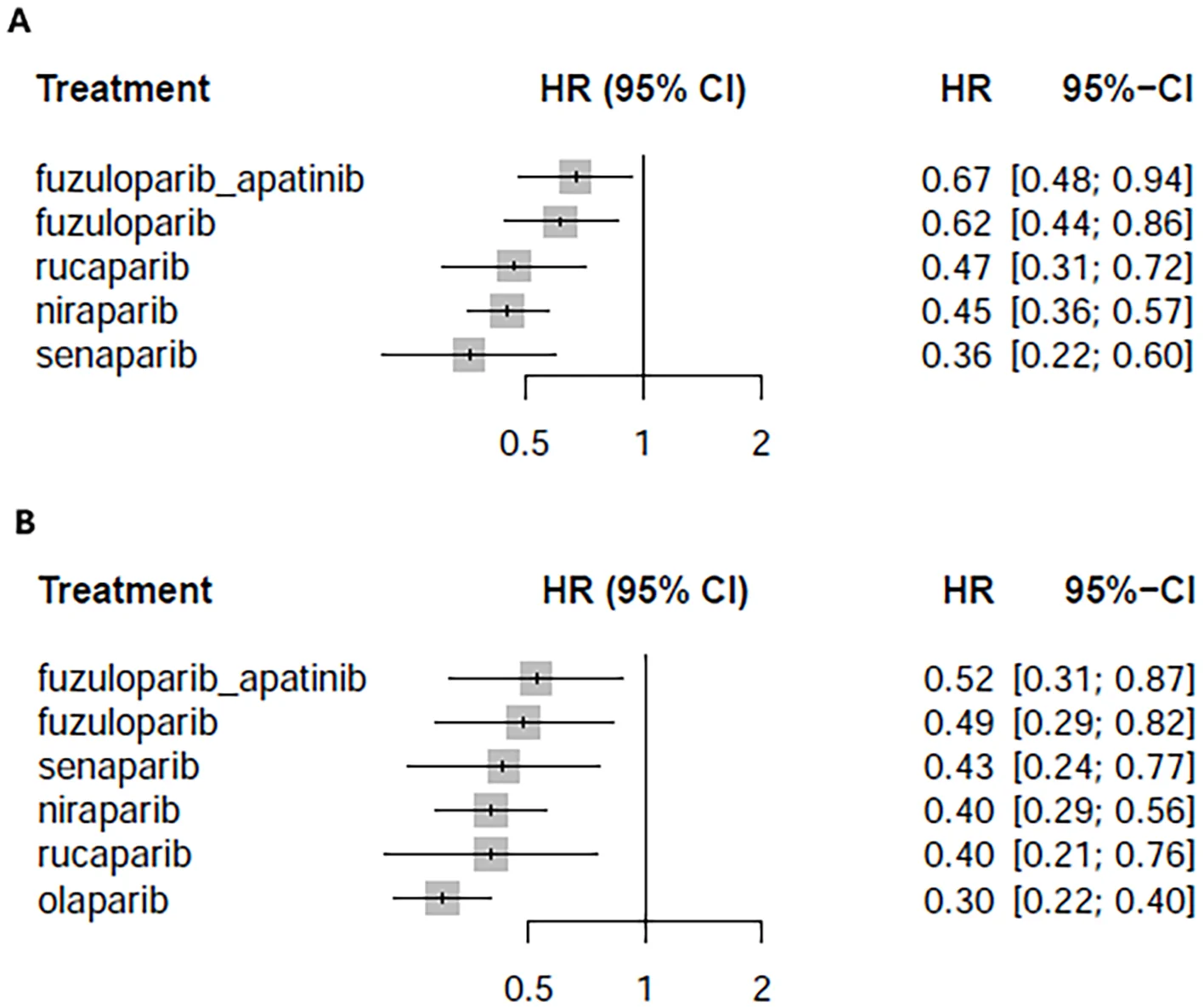
Network meta-analysis of progression-free survival in biomarker-defined populations. Forest plots of hazard ratios for progression-free survival in the **(A)** HRD-positive population and **(B)** BRCAm population. Hazard ratios are shown relative to placebo. Squares indicate point estimates, and horizontal lines indicate 95% confidence intervals.

Among active regimens, no statistically significant difference was observed between fuzuloparib plus apatinib and fuzuloparib monotherapy (network HR = 1.31; 95% CI, 0.77–2.24). However, this result should be interpreted as an absence of conclusive evidence for an incremental PFS benefit within the connected network, rather than as evidence that the combination provides no additional benefit. This comparison was largely informed by FZOCUS-1, which evaluated fuzuloparib plus apatinib; the limited adoption of this regimen outside selected geographic settings may restrict the generalizability of this finding to other PARP inhibitor–antiangiogenic combinations. The indirect comparison favored senaparib over fuzuloparib plus apatinib (HR = 1.87; 95% CI, 1.02–3.43). Because most active-regimen comparisons were informed indirectly through placebo and the network was sparse, these between-treatment differences should be interpreted cautiously.

Overall, the HRD-positive population showed a consistent and clinically meaningful PFS benefit from PARP inhibitor-based maintenance compared with placebo. However, the available connected-network evidence was insufficient to determine whether adding antiangiogenic therapy provides an incremental benefit over PARP inhibitor monotherapy. This finding should not be interpreted as evidence against the clinical value of PARP inhibitor–antiangiogenic combination maintenance strategies in general.

#### 3.4.2 BRCA-mutated population

In the BRCAm population (**Figure 3B**), all PARP inhibitor-based regimens included in the connected network significantly prolonged PFS compared with placebo, with generally larger treatment effects than those observed in broader populations. Olaparib showed the most favorable point estimate versus placebo (HR = 0.30; 95% CI, 0.22–0.40), followed by niraparib and rucaparib, both with HRs of approximately 0.40. Senaparib, fuzuloparib, and fuzuloparib plus apatinib also significantly improved PFS versus placebo, with HRs of 0.43, 0.49, and 0.52, respectively. According to P-scores, olaparib ranked highest, followed by niraparib, rucaparib, senaparib, fuzuloparib, fuzuloparib plus apatinib, and placebo.

Despite these numerical differences, indirect comparisons among active regimens did not identify statistically significant differences. In particular, fuzuloparib plus apatinib did not significantly improve PFS compared with fuzuloparib monotherapy (HR = 1.31; 95% CI, 0.71–2.41). Likewise, although olaparib had the most favorable point estimate, no statistically significant superiority was demonstrated over the other active regimens in the network.

Taken together, the BRCAm population derived the greatest magnitude of PFS benefit from PARP inhibitor-based maintenance, but the connected evidence did not establish clear superiority of one active regimen over another.

### 3.5 Secondary efficacy outcomes

#### 3.5.1 HRD-positive non-BRCA-mutated population

In the HRD-positive non-BRCAm population (**Figure 4A**), niraparib, rucaparib, and senaparib all significantly improved PFS versus placebo, with HRs of 0.50 (95% CI, 0.31–0.82), 0.46 (95% CI, 0.26–0.81), and 0.30 (95% CI, 0.15–0.60), respectively. No statistically significant differences were observed among the active regimens. No PARP inhibitor plus anti-angiogenic regimen was represented in this network, precluding assessment of combination maintenance in this subgroup.

**Figure 4:**
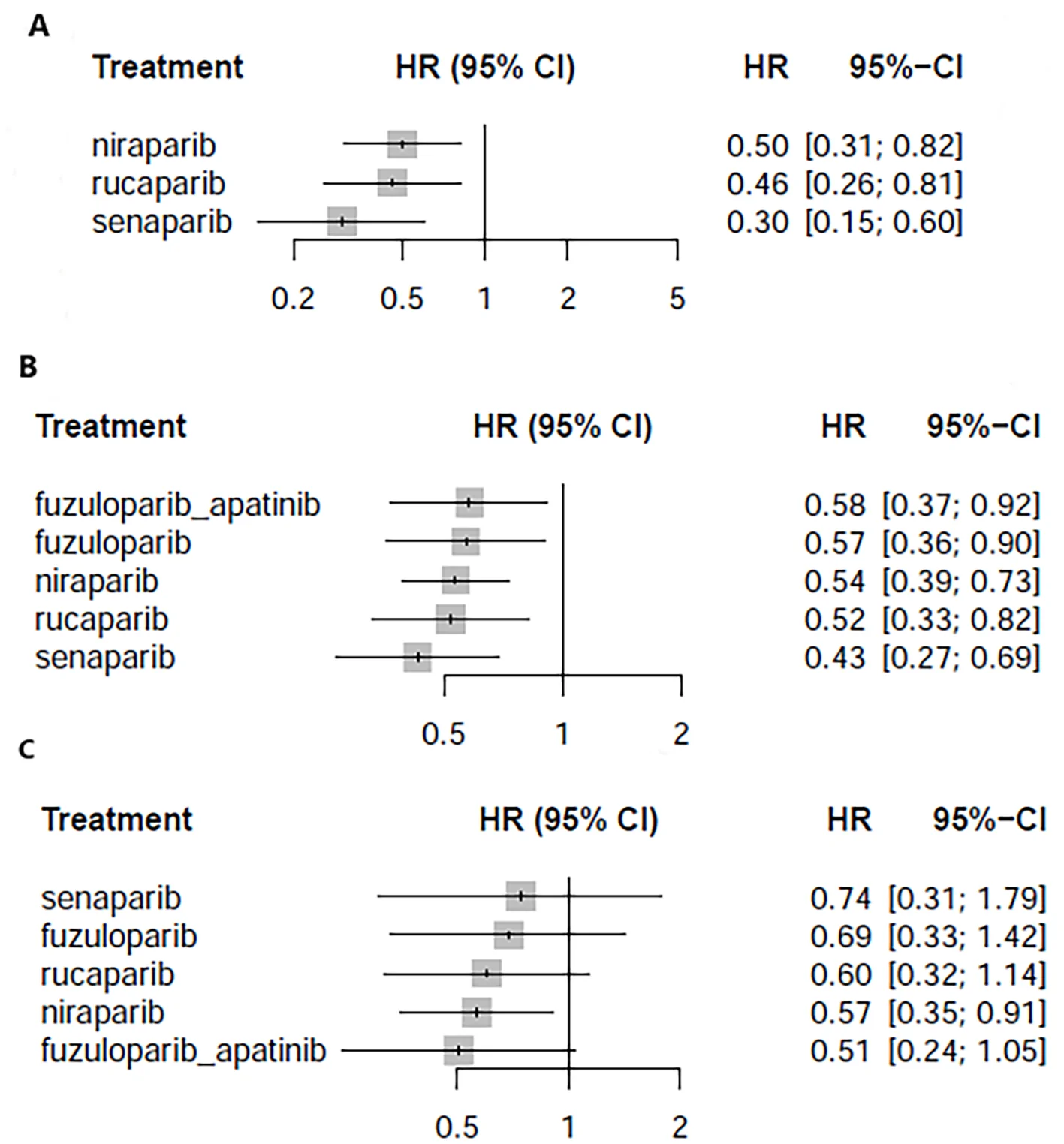
Network meta-analysis of progression-free survival in secondary populations. Forest plots of hazard ratios for progression-free survival in the **(A)** HRD-positive non-BRCAm population and **(B)** ITT population**(C)** HRP populations. Hazard ratios are shown relative to placebo.

#### 3.5.2 Intention-to-treat population

In the ITT population (**Figure 4B**), all PARP inhibitor-based regimens significantly improved PFS versus placebo, with HRs ranging from 0.43 to 0.58. No statistically significant differences were observed among the active regimens, including between fuzuloparib plus apatinib and fuzuloparib monotherapy (HR = 0.98; 95% CI, 0.63–1.51). Overall, treatment effects in the ITT population were smaller than those in the biomarker-selected populations.

#### 3.5.3 HRP population

In this subgroup (**Figure 4C**), niraparib was the only regimen that significantly improved PFS versus placebo (HR = 0.57; 95% CI, 0.35–0.91), whereas fuzuloparib plus apatinib showed the most favorable point estimate but did not reach statistical significance (HR = 0.51; 95% CI, 0.24–1.05). No active regimen demonstrated significant superiority over another, and the wide confidence intervals indicate that these findings should be interpreted cautiously.

To facilitate clinical interpretation of the relative effects estimated in the network meta-analysis, trial-level median PFS values in the treatment and control arms, together with within-trial median PFS differences, are summarized in **Supplementary Table S2**. Across trials, median PFS generally favored PARP inhibitor-based maintenance over placebo; however, the magnitude of the observed median differences varied across molecular subgroups, treatment regimens, and trial-specific control-arm prognosis. These data are presented descriptively and were not pooled, and therefore should not be interpreted as network-derived absolute treatment effects or compared directly across trials. In particular, hazard ratios cannot be directly translated into median PFS gains.

### 3.6 Exploratory effect-modification analysis by frontline bevacizumab background

Given the clinical relevance of bevacizumab in frontline treatment selection, we conducted an exploratory study-level analysis to assess whether frontline bevacizumab background modified the PFS benefit associated with PARP inhibitor-based maintenance therapy. Trials were categorized as requiring bevacizumab, allowing bevacizumab, or not allowing bevacizumab in the frontline setting.

Across all populations, PARP inhibitor-containing regimens were associated with improved PFS in all three categories, with pooled HRs of 0.49 in the required-bevacizumab group (95% CI, 0.32–0.75; I² = 88.9%), 0.56 in the allowed-bevacizumab group (95% CI, 0.50–0.62; I² = 0%), and 0.45 in the not-allowed-bevacizumab group (95% CI, 0.40–0.52; I² = 48.3%). In the overall meta-regression, bevacizumab background was not identified as a statistically significant effect modifier, with consistent results observed in both categorical and continuous bevacizumab-score models.

In molecular subgroup analyses (**Figure 5**), significant interaction signals were observed in the HRD-positive population (omnibus p = 0.0033) and the HRP population (omnibus p = 0.0209), but not in the ITT, BRCAm, or HRD-positive non-BRCAm populations. Given the exploratory study-level design and substantial heterogeneity in the required-bevacizumab category, these findings should be interpreted as hypothesis-generating rather than definitive.

**Figure 5:**
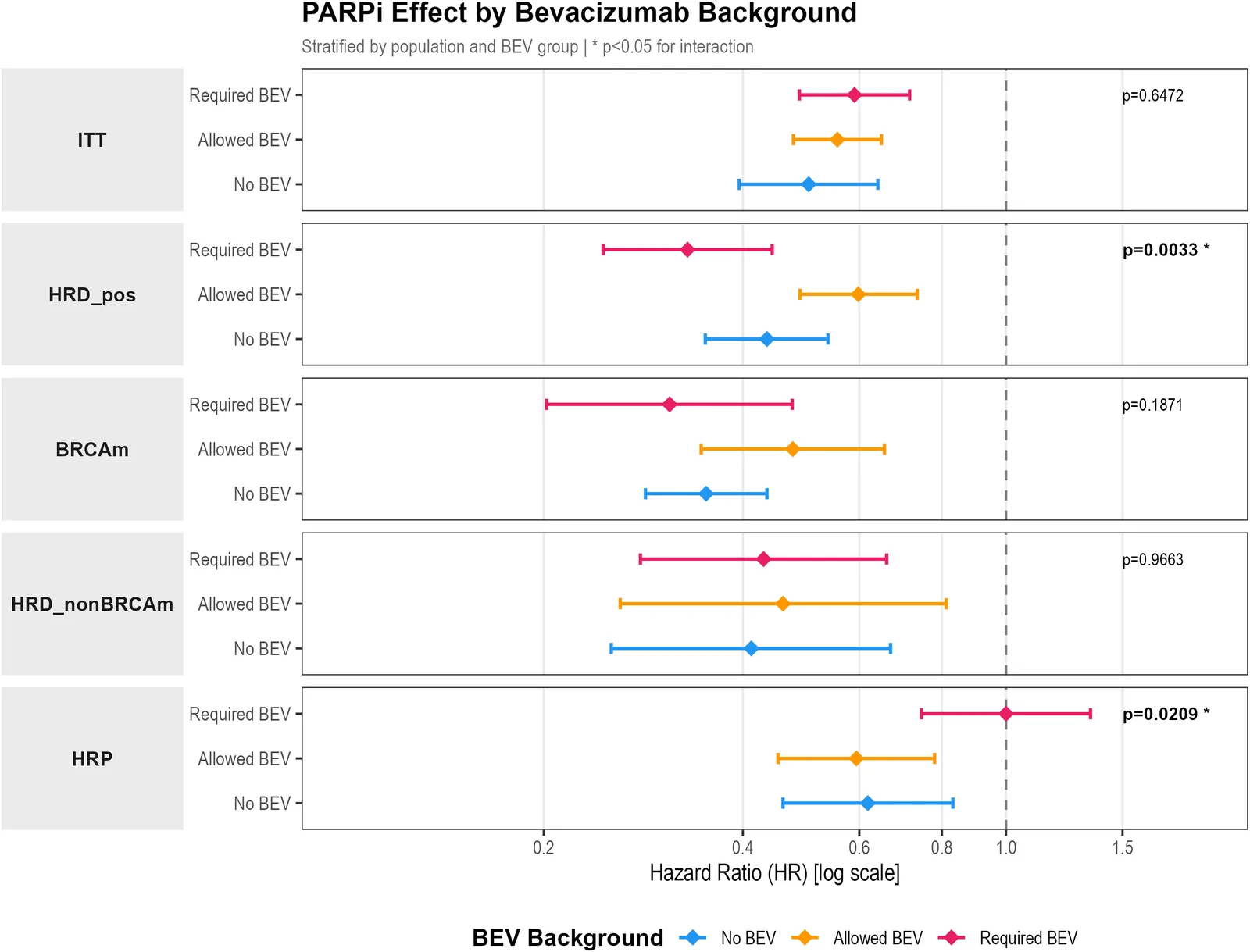
PARP inhibitor effect by frontline bevacizumab background. Pooled hazard ratios for progression-free survival with PARP inhibitor-containing maintenance therapy are shown according to frontline bevacizumab background and molecular population. Trials were classified as requiring bevacizumab, allowing bevacizumab, or not allowing bevacizumab. Diamonds represent pooled hazard ratios, and horizontal bars represent 95% confidence intervals. Hazard ratios below 1 favor PARP inhibitor-containing maintenance therapy. Interaction p values were obtained from study-level meta-regression models. Significant interaction signals were observed in the HRD-positive and HRP populations.

### 3.7 Sensitivity analyses

#### 3.7.1 Impact of including versus excluding FZOCUS-1

To assess the influence of FZOCUS-1 on the pooled estimates, we compared analyses including and excluding this trial. Inclusion of FZOCUS-1 expanded the treatment network by adding fuzuloparib-based regimens and introduced the only direct active-treatment comparison within the connected networks. However, pooled PFS estimates for PARP inhibitor-containing maintenance therapy versus placebo remained broadly unchanged across biomarker-defined populations when FZOCUS-1 was included versus excluded, supporting the robustness of the main findings (**Supplementary Figure S2**). The direct comparison of fuzuloparib plus apatinib versus fuzuloparib monotherapy did not provide statistically conclusive evidence of incremental benefit and was considered exploratory.

#### 3.7.2 Regimen-specific analysis across individual PARP inhibitors

Regimen-specific sensitivity analyses of individual PARP inhibitor monotherapies were broadly consistent with the main network findings (**Supplementary Figure S3**). Across the ITT, HRD-positive, BRCAm, and HRD-positive non-BRCAm populations, most evaluated PARP inhibitors showed improved PFS versus placebo. In contrast, treatment effects in the HRP population were less consistent, with niraparib being the only regimen showing a statistically significant PFS benefit versus placebo. Indirect comparisons among active PARP inhibitors did not identify statistically significant differences, suggesting that the overall findings were not driven by a single agent.

### 3.8 Safety outcomes

In the safety network meta-analysis versus placebo, all PARP inhibitor monotherapies significantly increased the risk of grade ≥3 adverse events (**Figure 6**). The estimated relative risks were 6.22 (95% CI, 3.38–11.45) for fuzuloparib, 3.50 (95% CI, 2.81–4.36) for niraparib, 3.27 (95% CI, 2.31–4.62) for senaparib, 2.66 (95% CI, 1.87–3.79) for rucaparib, and 2.12 (95% CI, 1.44–3.14) for olaparib.

**Figure 6:**
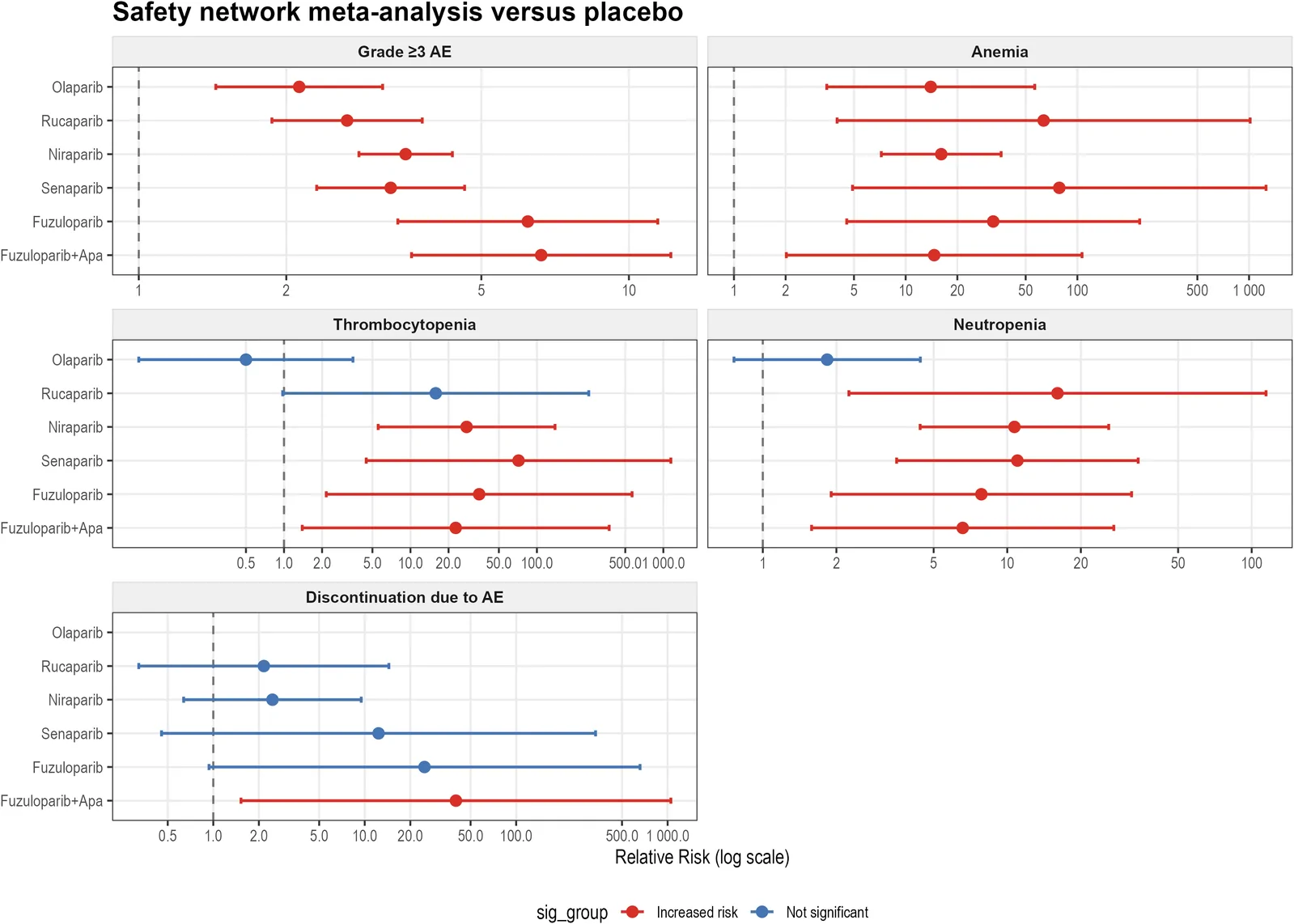
Safety network meta-analysis versus placebo. Relative risks and 95% confidence intervals are shown for grade ≥3 adverse events, grade ≥3 anemia, grade ≥3 thrombocytopenia, grade ≥3 neutropenia, and discontinuation due to adverse events. Estimates were derived from network meta-analysis versus placebo on a logarithmic scale. The dashed vertical line indicates RR = 1.0; values greater than 1 indicate increased risk relative to placebo. Red symbols denote statistically significant increased risk, whereas blue symbols indicate non-significant differences.

For selected hematologic toxicities, most regimens were associated with increased risks of grade ≥3 anemia, thrombocytopenia, and neutropenia relative to placebo. Olaparib, however, did not show a statistically significant increase in grade ≥3 thrombocytopenia or neutropenia, suggesting a comparatively more favorable hematologic safety profile among the evaluated monotherapies.

Data on discontinuation due to adverse events were limited and heterogeneous, with substantial between-study inconsistency (I² = 80.6%) and wide confidence intervals, limiting reliable comparative interpretation. Safety data for combination regimens were also sparse and not consistently connected within the safety network; therefore, comparative safety between PARP inhibitor monotherapy and PARP inhibitor–anti-angiogenic combination regimens remains uncertain. In the descriptive summary of PAOLA-1, olaparib plus bevacizumab was associated with higher rates of selected adverse events compared with bevacizumab alone; however, these data were not incorporated into the main safety NMA.

## 4 Discussion

In this updated network meta-analysis of first-line maintenance therapy for newly diagnosed advanced ovarian cancer, PARP inhibitor-based strategies significantly improved progression-free survival (PFS), with the most consistent and pronounced benefit observed in the BRCA-mutated (BRCAm) and homologous recombination deficiency (HRD)-positive populations. Benefit was also maintained in the intention-to-treat population, whereas results in homologous recombination proficient (HRP) disease were less consistent and should be interpreted cautiously. Across the connected networks, indirect comparisons did not demonstrate statistically significant superiority of one active PARP inhibitor-based regimen over another. In addition, although PARP inhibitor plus anti-angiogenic regimens showed activity versus placebo, their incremental benefit over PARP inhibitor monotherapy was not clearly established in most molecular subgroups.

These findings align with the current guideline emphasis on biomarker-directed selection of first-line maintenance therapy after response to platinum-based chemotherapy (14). Our results are broadly consistent with this framework, showing the greatest and most consistent PFS benefit in BRCAm and HRD-positive disease, with less certain benefit in HRP disease. By integrating evidence across available first-line maintenance trials, this analysis complements pivotal trial data and helps contextualize treatment selection when direct head-to-head comparisons are lacking.

The stronger benefit observed in BRCAm and HRD-positive populations is biologically plausible. PARP inhibition exploits defects in homologous recombination repair through synthetic lethality, and tumors with BRCA mutations or broader HRD features are particularly sensitive to this mechanism (6, 7). Importantly, benefit was not confined to BRCA-mutated disease: in the HRD-positive non-BRCAm population, PARP inhibitor monotherapy also significantly improved PFS, supporting the predictive value of HRD beyond BRCA status alone. By contrast, HRP disease remained the least certain subgroup, with smaller effect sizes, wider confidence intervals, and less consistent statistical significance. These findings support molecular stratification as a central principle of maintenance decision-making and suggest that in HRP disease, expected benefit should be weighed carefully against toxicity, treatment burden, cost, and patient preference.

An additional clinically relevant finding was the exploratory assessment of frontline bevacizumab background. This question is increasingly important because bevacizumab has been incorporated into first-line treatment and maintenance strategies in advanced ovarian cancer, as shown in GOG-0218 (12) and ICON7 (13), and prior bevacizumab exposure may influence maintenance choice and complicate interpretation of evidence from bevacizumab-background trials such as PAOLA-1 (9). In our study-level analyses, bevacizumab background was not identified as a statistically significant overall effect modifier, although subgroup-specific signals were observed in the HRD-positive and HRP populations. These findings should be considered hypothesis-generating, but they suggest that prior bevacizumab exposure alone may not fully explain differences in the relative benefit of PARP inhibitor-based maintenance across all molecular groups.

The role of combining PARP inhibition with anti-angiogenic therapy remains unresolved (11). Although this strategy is biologically plausible, the present analysis did not show a statistically confirmed incremental PFS benefit for combination therapy over PARP inhibitor monotherapy in the connected subgroup networks. This was particularly notable after inclusion of FZOCUS-1, which contributed the only direct active-treatment comparison within the connected evidence structure; even in that setting, the addition of apatinib to fuzuloparib did not demonstrate a clear incremental advantage. Taken together, these findings do not establish a clear incremental benefit for fuzuloparib plus apatinib over fuzuloparib monotherapy within the connected network. This result should not be extrapolated to exclude the potential clinical value of other PARP inhibitor–anti-angiogenic combinations, including olaparib plus bevacizumab evaluated in PAOLA-1, which was not included in the connected network.

Another important observation was the absence of statistically significant differences among active PARP inhibitors in indirect comparisons. Although some regimens showed numerically favorable point estimates or rankings in specific subgroups, these differences were not supported by statistically significant network estimates. This suggests that comparative efficacy differences among currently available PARP inhibitor-based maintenance regimens remain uncertain. In practice, treatment selection among these agents may therefore depend more on toxicity profile, dosing schedule, regulatory approval, availability, prior bevacizumab exposure, and patient preference than on any proven superiority of one active regimen over another.

Safety remains particularly important in the maintenance setting. All PARP inhibitor monotherapies increased the risk of grade ≥3 adverse events versus placebo (23), and olaparib appeared to have a comparatively more favorable hematologic safety profile among the evaluated monotherapies. However, comparative safety assessment of combination regimens remained limited because safety data were incompletely connected and variably reported across trials. Accordingly, while combination treatment may increase treatment burden, comparative safety conclusions between PARP inhibitor monotherapy and PARP inhibitor–anti-angiogenic combinations should remain cautious.

The predominance of placebo-controlled trials in the connected networks provided a common reference that facilitated indirect comparisons across active regimens—a methodological strength of this analysis. However several limitations should be acknowledged. First, many evidence networks were sparse and predominantly placebo-centered, with few closed loops and limited direct comparisons between active PARP inhibitor-based regimens. As a result, formal assessment of inconsistency and reliable estimation of between-study heterogeneity were limited in several molecular subgroup networks. Therefore, indirect comparisons and treatment rankings should be interpreted cautiously. Second, the exploratory bevacizumab-background analysis was conducted at the study level and was therefore susceptible to ecological bias. Third, transitivity may have been influenced by differences in HRD assays and definitions (24), bevacizumab exposure, treatment duration, follow-up, and dose-modification rules across trials. Fourth, the network meta-analysis estimated relative treatment effects, expressed as HRs, rather than pooled absolute PFS gains. Although the common placebo-controlled design of most included trials provided a relatively consistent reference for indirect comparisons, control-arm median PFS varied across studies and molecular subgroups. We therefore summarized trial-level median PFS and within-trial median differences descriptively (**Supplementary Table S2**), rather than deriving or pooling absolute PFS effects from HRs. HRs cannot be directly converted into median PFS gains, and cross-trial comparisons of median PFS may be confounded by differences in patient populations, baseline prognosis, and trial design. Finally, overall survival data were not mature or consistently available across studies (25, 26), so the present analysis primarily informs PFS-based decision-making.

Despite these limitations, this study provides an updated comparative synthesis across clinically relevant molecular subgroups and incorporates newer evidence, including FZOCUS-1, which expands the available network for fuzuloparib-based maintenance therapy. The results reinforce the central role of biomarker-directed selection in first-line maintenance treatment for newly diagnosed advanced ovarian cancer and highlight the need for more direct comparative trials, particularly between active PARP inhibitor-based regimens and between monotherapy and combination strategies.

In conclusion, this updated network meta-analysis supports biomarker-directed PARP inhibitor-based first-line maintenance therapy for newly diagnosed advanced ovarian cancer, with the greatest and most consistent PFS benefit observed in BRCAm and HRD-positive disease. Current evidence does not establish clear superiority among active PARP inhibitor-based regimens, and the incremental benefit of adding anti-angiogenic therapy to PARP inhibition remains uncertain because connected-network evidence was limited and largely informed by FZOCUS-1; this finding should not be interpreted as evidence against the clinical value of PARP inhibitor–antiangiogenic combination maintenance strategies in general, including olaparib plus bevacizumab. Maintenance selection should be individualized according to molecular status, prior bevacizumab exposure, expected benefit, safety, treatment availability, and patient preference.
