# Supplementary material for: PARP inhibitor-based first-line maintenance therapy for newly diagnosed advanced ovarian cancer across molecular subgroups: an updated systematic review and network meta-analysis
Source: Front Oncol. 2026 Sep 18;16:1884407. doi: 10.3389/fonc.2026.1884407 (PMC13630527; doi:10.3389/fonc.2026.1884407)
Supplement: Supplementary file 1 [file Table1.docx]

**Supplementary Table S1. Search strategies used for each database**

| **Database/source** | **Search strategy** |
| --- | --- |
| **PubMed /MEDLINE** | (("Ovarian Neoplasms"[Mesh] OR "Fallopian Tube Neoplasms"[Mesh] OR "Peritoneal Neoplasms"[Mesh] OR ovarian cancer*[tiab] OR ovarian carcinoma*[tiab] OR ovarian neoplasm*[tiab] OR epithelial ovarian[tiab] OR fallopian tube cancer*[tiab] OR fallopian tube carcinoma*[tiab] OR fallopian tube neoplasm*[tiab] OR primary peritoneal cancer*[tiab] OR primary peritoneal carcinoma*[tiab] OR primary peritoneal neoplasm*[tiab]) AND ("Poly(ADP-ribose) Polymerase Inhibitors"[Mesh] OR PARP inhibitor*[tiab] OR PARP-inhibitor*[tiab] OR poly ADP ribose polymerase inhibitor*[tiab] OR poly(ADP-ribose) polymerase inhibitor*[tiab] OR olaparib[tiab] OR Lynparza[tiab] OR niraparib[tiab] OR Zejula[tiab] OR rucaparib[tiab] OR Rubraca[tiab] OR fuzuloparib[tiab] OR fluzoparib[tiab] OR senaparib[tiab]) AND (maintenance[tiab] OR "maintenance therapy"[tiab] OR first-line[tiab] OR frontline[tiab] OR front-line[tiab] OR newly diagnosed[tiab] OR platinum-based[tiab] OR platinum based[tiab] OR response to platinum[tiab] OR after chemotherapy[tiab]) AND (randomized[tiab] OR randomised[tiab] OR placebo[tiab] OR double-blind[tiab] OR "double blind"[tiab] OR "phase 3"[tiab] OR "phase III"[tiab] OR "Randomized Controlled Trial"[Publication Type] OR "Clinical Trial, Phase III"[Publication Type])) AND ("1900/01/01"[Date - Publication] : "2026/04/01"[Date - Publication]) |
| **PubMed/MEDLINE supplementary trial-name search** | ((SOLO-1[tiab] OR SOLO1[tiab] OR PAOLA-1[tiab] OR PAOLA1[tiab] OR PRIMA[tiab] OR ATHENA-MONO[tiab] OR "ATHENA MONO"[tiab] OR PRIME[tiab] OR FLAMES[tiab] OR FZOCUS-1[tiab] OR FZOCUS1[tiab]) AND (ovarian[tiab] OR fallopian[tiab] OR peritoneal[tiab])) AND ("1900/01/01"[Date - Publication] : "2026/04/01"[Date - Publication]) |
| **Embase via Ovid** | (exp ovary cancer/ OR exp ovary tumor/ OR exp fallopian tube cancer/ OR exp fallopian tube tumor/ OR exp peritoneum cancer/ OR exp peritoneum tumor/ OR (ovarian adj3 (cancer* or carcinoma* or neoplasm* or tumor* or tumour* or malignan*)). ti,ab,kw. OR (epithelial adj3 ovarian). ti,ab,kw. OR (fallopian adj3 tube adj3 (cancer* or carcinoma* or neoplasm* or tumor* or tumour* or malignan*). ti,ab,kw. OR (primary adj3 peritoneal adj3 (cancer* or carcinoma* or neoplasm* or tumor* or tumour* or malignan*). ti,ab,kw.) AND (exp poly adenosine diphosphate ribose polymerase inhibitor/ OR exp olaparib/ OR exp niraparib/ OR exp rucaparib/ OR (PARP adj3 inhibitor*). ti,ab,kw. OR PARP-inhibitor*. ti,ab,kw. OR (poly ADP ribose polymerase adj3 inhibitor*). ti,ab,kw. OR (polyadenosine diphosphate ribose polymerase adj3 inhibitor*). ti,ab,kw. OR olaparib.ti,ab,kw. OR Lynparza.ti,ab,kw. OR niraparib.ti,ab,kw. OR Zejula.ti,ab,kw. OR rucaparib.ti,ab,kw. OR Rubraca.ti,ab,kw. OR fuzuloparib.ti,ab,kw. OR fluzoparib.ti,ab,kw. OR senaparib.ti,ab,kw.) AND (maintenance.ti,ab,kw. OR "maintenance therapy”. ti,ab,kw. OR first-line.ti,ab,kw. OR frontline.ti,ab,kw. OR front-line.ti,ab,kw. OR newly diagnosed.ti,ab,kw. OR platinum-based.ti,ab,kw. OR "platinum based”. ti,ab,kw. OR "response to platinum”. ti,ab,kw. OR "after chemotherapy”. ti,ab,kw.) AND (randomized.ti,ab,kw. OR randomised.ti,ab,kw. OR placebo.ti,ab,kw. OR double-blind.ti,ab,kw. OR "double blind”. ti,ab,kw. OR phase 3. ti,ab,kw. OR phase III.ti,ab,kw. OR exp randomized controlled trial/ OR exp phase 3 clinical trial/) |
| **Embase supplementary trial-name search** | ((SOLO-1 OR SOLO1 OR PAOLA-1 OR PAOLA1 OR PRIMA OR ATHENA-MONO OR "ATHENA MONO" OR PRIME OR FLAMES OR FZOCUS-1 OR FZOCUS1). ti,ab,kw.) AND (ovarian.ti,ab,kw. OR fallopian.ti,ab,kw. OR peritoneal.ti,ab,kw.) |
| **Web of Science** | TS=((("ovarian cancer*" OR "ovarian carcinoma*" OR "ovarian neoplasm*" OR "epithelial ovarian" OR "fallopian tube cancer*" OR "fallopian tube carcinoma*" OR "fallopian tube neoplasm*" OR "primary peritoneal cancer*" OR "primary peritoneal carcinoma*" OR "primary peritoneal neoplasm*") AND ("PARP inhibitor*" OR "PARP-inhibitor*" OR "poly ADP ribose polymerase inhibitor*" OR "poly(ADP-ribose) polymerase inhibitor*" OR olaparib OR Lynparza OR niraparib OR Zejula OR rucaparib OR Rubraca OR fuzuloparib OR fluzoparib OR senaparib) AND (maintenance OR "maintenance therapy" OR "first-line" OR frontline OR "front-line" OR "newly diagnosed" OR "platinum-based" OR "platinum based" OR "response to platinum" OR "after chemotherapy") AND (randomized OR randomised OR placebo OR "double-blind" OR "double blind" OR "phase 3" OR "phase III"))) |
| **Web of Science supplementary trial-name search** | TS = ((("SOLO-1" OR SOLO1 OR "PAOLA-1" OR PAOLA1 OR PRIMA OR "ATHENA-MONO" OR "ATHENA MONO" OR PRIME OR FLAMES OR "FZOCUS-1" OR FZOCUS1) AND (ovarian OR fallopian OR peritoneal))) |
| **ClinicalTrial.gov** | The condition terms "ovarian cancer", "fallopian tube cancer", and "primary peritoneal cancer" were searched separately. Each condition term was combined with the following intervention or other terms: "PARP inhibitor”, “olaparib", "niraparib", "rucaparib", "fuzuloparib", "fluzoparib", "senaparib", "bevacizumab olaparib", "SOLO-1", "PAOLA-1", "PRIMA", "ATHENA-MONO", "PRIME", "FLAMES", and "FZOCUS-1". Interventional studies were screened without restrictions on recruitment status or results availability. |
| **Conference proceedings** | ASCO, ESMO, SGO, and IGCS conference proceedings were manually searched from the earliest year available online to 1 April 2026. Search terms included ovarian cancer, fallopian tube cancer, primary peritoneal cancer, PARP inhibitor, olaparib, niraparib, rucaparib, fuzuloparib, fluzoparib, senaparib, bevacizumab, maintenance, first-line, frontline, newly diagnosed, platinum-based, randomized, phase 3, phase III, double-blind, SOLO-1, PAOLA-1, PRIMA, ATHENA-MONO, PRIME, FLAMES, and FZOCUS-1. |

**Figure S1. Risk of bias summary of the included randomized trials.**

Domains assessed by the Cochrane Risk of Bias 2 tool included randomization process (D1), deviations from intended interventions (D2), missing outcome data (D3), outcome measurement (D4), and selection of the reported result (D5). Green indicates low risk of bias.

**Figure S2. Impact of including versus excluding FZOCUS-1 on pooled PFS estimates.**

Pooled hazard ratios and 95% confidence intervals for PARP inhibitor-containing maintenance therapy versus placebo are shown with and without inclusion of FZOCUS-1 across biomarker-defined populations. The dashed line indicates HR = 1.0. Estimates were broadly similar in the two analyses.

**Figure S3. Regimen-specific sensitivity analysis of individual PARP inhibitor monotherapy regimens.**

Forest plots show progression-free survival hazard ratios and 95% confidence intervals for available PARP inhibitor monotherapy regimens compared with placebo across the ITT population and biomarker-defined subgroups. Estimates were derived from random-effects network meta-analysis. The dashed vertical line indicates HR = 1.0, with values below 1.0 favoring PARP inhibitor maintenance therapy. Regimen-specific estimates were generally consistent with the primary analysis, suggesting that the overall results were not driven by a single PARP inhibitor.

**Supplementary Table S2. Trial-level median progression-free survival and within-trial median differences reported in randomized controlled trials of first-line maintenance therapy**

| **Trial** | **Population/subgroup** | **Treatment** | **Control** | **Median PFS, treatment arm, months (95% CI)** | **Median PFS, control arm, months (95% CI)** | **Within-trial median PFS difference, months** | **HR (95% CI)** |
| --- | --- | --- | --- | --- | --- | --- | --- |
| SOLO-1 | BRCAm | Olaparib | Placebo | NR (41.9–NR) | 13.8 (11.1–19.3) | NA | 0.30 (0.23–0.41) |
| PAOLA-1† | ITT | Olaparib + bevacizumab | Placebo + bevacizumab | 22.1 (20.3–23.7) | 16.6 (14.8–19.1) | 5.5 | 0.59 (0.49–0.72) |
| PAOLA-1† | HRD-positive | Olaparib + bevacizumab | Placebo + bevacizumab | 37.2 (31.0–NR) | 17.7 (14.7–24.6) | 19.5 | 0.33 (0.25–0.45) |
| PAOLA-1† | BRCAm | Olaparib + bevacizumab | Placebo + bevacizumab | 46.1 (36.8–NR) | 24.7 (17.2–NR) | 21.4 | 0.31 (0.20–0.47) |
| PAOLA-1† | HRD-positive, non-BRCAm | Olaparib + bevacizumab | Placebo + bevacizumab | 31.0 (22.9–NR) | 13.0 (9.9–17.1) | 18.0 | 0.43 (0.28–0.66) |
| PAOLA-1† | HRP | Olaparib + bevacizumab | Placebo + bevacizumab | 17.3 (16.7–19.0) | 16.0 (14.6–18.0) | 1.3 | 0.92 (0.72–1.17) |
| PRIMA | ITT | Niraparib | Placebo | 13.8 (11.5–15.2) | 8.2 (6.8–9.6) | 5.6 | 0.62 (0.50–0.76) |
| PRIMA | HRD-positive | Niraparib | Placebo | 21.9 (18.7–NR) | 10.4 (8.4–12.9) | 11.5 | 0.43 (0.31–0.59) |
| PRIMA | BRCAm | Niraparib | Placebo | 22.1 (18.6–NR) | 10.9 (7.2–17.0) | 11.2 | 0.40 (0.27–0.62) |
| PRIMA | HRD-positive, non-BRCAm | Niraparib | Placebo | 19.6 (16.0–NR) | 8.2 (5.7–12.2) | 11.4 | 0.50 (0.31–0.83) |
| PRIMA | HRP | Niraparib | Placebo | 8.1 (6.9–9.3) | 5.8 (5.4–7.2) | 2.3 | 0.68 (0.49–0.94) |
| ATHENA-MONO | ITT | Rucaparib | Placebo | 20.2 (15.2–24.7) | 9.2 (8.3–12.2) | 11.0 | 0.52 (0.40–0.68) |
| ATHENA-MONO | HRD-positive | Rucaparib | Placebo | 28.7 (23.0–NR) | 11.3 (9.1–22.1) | 17.4 | 0.47 (0.31–0.72) |
| ATHENA-MONO | BRCAm | Rucaparib | Placebo | 35.8 (27.6–NR) | 12.0 (5.6–21.3) | 23.8 | 0.40 (0.21–0.75) |
| ATHENA-MONO | HRD-positive, non-BRCAm | Rucaparib | Placebo | 20.3 (14.6–NR) | 9.2 (5.8–13.4) | 11.1 | 0.46 (0.26–0.80) |
| ATHENA-MONO | HRP | Rucaparib | Placebo | 12.1 (11.1–17.7) | 9.1 (4.0–12.2) | 3.0 | 0.65 (0.45–0.95) |
| PRIME | ITT | Niraparib | Placebo | 24.8 (19.2–NE) | 8.3 (7.3–11.1) | 16.5 | 0.45 (0.34–0.60) |
| PRIME | HRD-positive | Niraparib | Placebo | NR (22.3–NE) | 11.0 (8.3–13.8) | NA | 0.48 (0.34–0.68) |
| PRIME | BRCAm | Niraparib | Placebo | NR (NE–NE) | 10.8 (NE–NE) | NA | 0.40 (0.23–0.68) |
| PRIME | HRP | Niraparib | Placebo | 16.6 (NE–NE) | 5.5 (NE–NE) | 11.1 | 0.41 (0.22–0.75) |
| FLAMES | ITT | Senaparib | Placebo | NR (26.3–NR) | 13.6 (10.9–16.6) | NA | 0.43 (0.32–0.58) |
| FLAMES | HRD-positive | Senaparib | Placebo | NR (NE–NE) | 15.7 (11.0–21.3) | NA | 0.36 (0.22–0.60) |
| FLAMES | BRCAm | Senaparib | Placebo | NR (NE–NE) | 15.6 (11.0–21.4) | NA | 0.43 (0.24–0.76) |
| FLAMES | HRD-positive, non-BRCAm | Senaparib | Placebo | NR (NE–NE) | 12.9 (8.3–21.3) | NA | 0.30 (0.15–0.60) |
| FLAMES | HRP | Senaparib | Placebo | 27.1 (8.4–NR) | 19.5 (7.8–NR) | 7.6 | 0.74 (0.36–1.54) |
| FZOCUS-1 | ITT | Fuzuloparib + apatinib | Placebo | 26.9 (20.3–36.6) | 11.1 (8.3–16.6) | 15.8 | 0.57 (0.44–0.75) |
| FZOCUS-1 | ITT | Fuzuloparib | Placebo | 29.9 (22.1–36.1) | 11.1 (8.3–16.6) | 18.8 | 0.58 (0.44–0.75) |
| FZOCUS-1 | HRD-positive | Fuzuloparib + apatinib | Placebo | 34.1 (22.4–41.2) | 20.3 (14.8–27.7) | 13.8 | 0.67 (0.42–1.06) |
| FZOCUS-1 | HRD-positive | Fuzuloparib | Placebo | 35.8 (27.3–NR) | 20.3 (14.8–27.7) | 15.5 | 0.62 (0.39–0.98) |
| FZOCUS-1 | BRCAm | Fuzuloparib + apatinib | Placebo | NR (NE–NE) | NR (NE–NE) | NA | 0.52 (0.25–1.07) |
| FZOCUS-1 | BRCAm | Fuzuloparib | Placebo | NR (NE–NE) | NR (NE–NE) | NA | 0.49 (0.23–1.02) |
| FZOCUS-1 | HRP | Fuzuloparib + apatinib | Placebo | 16.6 (10.2–22.1) | 11.0 (6.5–16.6) | 5.6 | 0.73 (0.45–1.19) |
| FZOCUS-1 | HRP | Fuzuloparib | Placebo | 11 (6.5–16.6) | 11.0 (6.5–16.6) | 0 | 0.55 (0.28–1.08) |

**Abbreviations:** BRCAm, BRCA-mutated; CI, confidence interval; HR, hazard ratio; HRD, homologous recombination deficiency; HRP, homologous recombination proficient; ITT, intention-to-treat; NA, not applicable because a within-trial median PFS difference could not be calculated; NE, not estimable; NR, not reached; PFS, progression-free survival.

**Notes:** Median PFS values and within-trial median PFS differences are descriptive, trial-specific outcomes reported in the original studies. The within-trial median PFS difference was calculated as the median PFS in the treatment arm minus that in the control arm. These outcomes were not pooled and should not be interpreted as network-estimated absolute treatment effects or compared directly across trials, because of differences in baseline prognosis, molecular subgroup composition, eligibility criteria, follow-up duration, and outcome-assessment procedures. Hazard ratios do not directly translate into median PFS differences.

† In PAOLA-1, both randomized groups received bevacizumab. Therefore, the reported median PFS difference represents the incremental effect of adding olaparib to bevacizumab, rather than the effect of olaparib plus bevacizumab versus placebo alone. PAOLA-1 was not included in the primary connected network because bevacizumab was administered in both treatment groups.
